# Overexpression of *Hevea brasiliensis HbCDS2* Gene Enhances Cold Tolerance in Transgenic *Arabidopsis*

**DOI:** 10.3390/plants14233591

**Published:** 2025-11-25

**Authors:** Wencai Yu, Guanghong Kong, Huajin Ya, Hanyao Zhang

**Affiliations:** 1 Yunnan Key Laboratory of Sustainable Utilization Research on Rubber Tree, National and Local Joint Engineering Research Center of Breeding and Cultivation Technology of Rubber Tree, Yunnan Institute of Tropical Crops, Jinghong 666100, China; kgh.003@163.com (G.K.); yahuajin@swfu.edu.cn (H.Y.); 2 Key Laboratory of Conservation and Utilization of Southwest Mountain Forest Resources, Ministry of Education, Southwest Forestry University, Kunming 650224, China

**Keywords:** rubber tree, superoxide dismutase, *HbCSD2*, cold resistance

## Abstract

Rubber tree (*Hevea brasiliensis*) is a crucial economic crop in tropical regions worldwide; however, low temperature in some areas have become a major source of abiotic stress that constrains the sustainable development of the natural rubber industry. Superoxide dismutase (SOD) is an enzyme that catalyzes the dismutation of the superoxide anion radical (O_2_•^−^) into oxygen (O_2_) and hydrogen peroxide (H_2_O_2_). Thus, SOD is an important antioxidant defense under plant stress defense, and also may help to improve rubber tree protection from the cold. In this study, a Cu/Zn superoxide dismutase gene, *HbCSD2*, was successfully cloned from the rubber tree via PCR amplification. Subcellular localization analysis revealed that *HbCSD2* is localized in the cytoplasm and nucleus. Under low-temperature stress, the seed germination rate, fresh weight, and survival rate of *HbCSD2*-overexpressing transgenic *Arabidopsis* were significantly higher than those of the wild-type (WT) plants. Conversely, the malformed seedling rate was considerably lower. Compared to WT plants, the transgenic *Arabidopsis* showed marked increases in SOD, catalase (CAT), and peroxidase (POD) activity, as well as the soluble sugar content. Meanwhile, the levels of malondialdehyde (MDA), H_2_O_2_, and O_2_•^−^ were significantly lower. This study confirms that *HbCSD2* enhances cold tolerance by boosting antioxidant enzyme activity and ROS scavenging capabilities, while reducing membrane lipid peroxidation. These findings offer valuable insights into the regulatory role of *HbCSD2* and the mechanisms behind stress responses in the rubber tree.

## 1. Introduction

Plants are frequently exposed to various abiotic stresses, including extreme temperatures, drought, waterlogging, high salinity, ultraviolet radiation, and heavy metal toxicity, during their growth, development, and production. These stresses often disrupt the homeostasis within plant cells, leading to the production of large amounts of reactive oxygen species (ROS) [1,2], including superoxide anions (O_2_•^−^), hydroxyl radicals (•OH), and hydrogen peroxide (H_2_O_2_), etc. [3,4,5]. Excessive accumulation of ROS in plant cells can cause membrane lipid peroxidation, structural damage to nucleic acids and proteins, and the disruption of carbohydrate synthesis and metabolism. In severe cases, it can lead to cell death, affecting plant growth and development, and ultimately reducing crop yield [6,7,8]. In order to adapt to adverse environmental conditions, plants have evolved complex and efficient antioxidant defense systems that effectively scavenge ROS and protect cells from damage [5]. Among them, superoxide dismutase (SOD), the first line of defense in the plant antioxidant enzyme defense system and one of the most important antioxidant enzymes in plants that can catalyze the O_2_•^−^ to produce O_2_ and H_2_O_2_, thereby effectively scavenging ROS [5,9,10]. SOD is a metal enzyme, and based on different metal cofactors (Cu, Zn, Mn, Fe, and Ni), the SOD family genes in higher plants are classified into three subfamilies, namely the Cu/Zn-SOD, Fe/Mn-SOD, and Ni-SOD subfamilies [11,12].

SOD plays a crucial role in enhancing plant tolerance to various abiotic stresses and has received significant research attention. Numerous studies have demonstrated that Cu/ZnSOD plays a crucial role in plant responses to abiotic stress. For example, drought stress significantly upregulated the expression of the *AhCuZnSOD* gene in *Arachis hypogaea*. Further investigation revealed that the activities of SOD, ascorbate peroxidase (APX), catalase (CAT), and glutathione reductase (GR) in the *AhCuZnSOD* gene transgenic tobacco lines under drought stress were greater than those in the wild-type (WT) lines. Concurrently, the levels of reactive oxygen species (H_2_O_2_ and O•^2−^) were substantially lower in the transgenic lines than in the WT plants. Moreover, the survival rate of the transgenic lines significantly improved under drought stress compared with the WT plants [13]. The expression of the *SaCu/ZnSOD* gene in *Sedum alfredii* is upregulated under cadmium (Cd) stress. The heterologous overexpression of *SaCu/ZnSOD* in *Arabidopsis* significantly increased the tolerance of transgenic plants to Cd stress [14]. The overexpression of *PagSOD2a* in transgenic poplar enhanced salt tolerance by increasing the expression of *CuZnSOD*, increasing SOD activity, and improving the capacity to scavenge O_2_•^−^ [15]. Under high-salt stress, heterologous expression of *Jatropha curcas JcCu/ZnSOD* in *Arabidopsis* significantly increased the SOD enzyme activity, decreased the malondialdehyde (MDA) content, and enhanced the tolerance of transgenic *Arabidopsis* compared with the WT plants [16]. Lin et al. [17] reported that the transfer of the *CmSOD* and *AtSOD* genes into *Arabidopsis* significantly enhanced the cold resistance of transgenic plants compared to the WT. In addition, the expression of the cold stress response-related gene *AtCBF2* and the drought stress response-related gene *AtRD29A/B* was activated in the transgenic lines. Therefore, overexpressing *SOD* functional genes through genetic engineering is a feasible strategy for developing high-resistance crop varieties and provides a novel approach for improving plant resistance traits.

The rubber tree (*Hevea brasiliensis*) is a perennial tropical rainforest species belonging to the Euphorbiaceae family and genus *Hevea* that is native to the Amazon Basin in South America [18,19]. In 1876, Wickham collected more than 70,000 rubber tree seeds from the Rio Tapajoz region of the upper Amazon and transported them to the Royal Botanic Garden (Kew Garden) for cultivation; these seedings were then sent to Southeast Asian countries for trial planting, marking the beginning of commercial rubber tree cultivation [20]. In recent years, with the development of the global economy, the demand for natural rubber has increased annually, and the rubber planting industry has continued to expand into nontraditional rubber tree planting areas, such as north-central Vietnam, northern India, southwestern China, the southern highlands of Brazil, and northeastern Thailand [21]. In these regions, rubber tree plantations are frequently affected by cold currents, leading to cold injury symptoms, such as reduced leaf area, leaf withering, shoot dieback, stem desiccation, and gum exudation, and in severe cases, even whole-plant mortality. Cold injury has become one of the primary natural disasters restricting the sustainable development of the natural rubber industry [22,23]. Therefore, identifying key functional genes associated with cold tolerance in the rubber tree is of significant practical importance for molecular breeding strategies aimed at improving its cold resistance.

Although Cu/Zn-SOD is widely involved in plant cold resistance, but its function and regulatory mechanism in response to low temperature stress in the rubber tree is still unclear. Our previous study found that nine *HbSOD* genes in the rubber tree genome and a *HbCu/ZnSOD2* (*HbCSD2*) was significantly up-regulated under low-temperature stress and was maintained at a high expression level [24]. At the same time, the promoter region contained a large number of stress- and hormone-related cis-acting elements [24]. In this study, we successfully cloned the full-length CDS sequence of the *HbCSD2* gene from the rubber tree and transformed it into *Arabidopsis* to evaluate its biological function and cold resistance during low-temperature stress. This study may provide a novel gene resource and a theoretical basis for improving the cold tolerance in high-yield rubber tree varieties through genetic engineering.

## 2. Results

### 2.1. Gene Cloning and Sequence Analysis of HbCSD2

Our previous study revealed that a *Cu/ZnSOD* (*HbCSD2*) gene was significantly upregulated under low-temperature stress and maintained at a high expression level. To further investigate the biological functions of the *HbCSD2* gene under low-temperature stress, we successfully cloned the full-length coding sequence (CDS) of *HbCSD2* from the rubber tree. The full-length CDS of *HbCSD2* contains 459 bp and encodes a predicted protein of 152 amino acids (Figure S1). Compared with the genomic HbCSD2 protein sequence (XP_021669573.1), the deduced amino acid sequence contains a minor deletion spanning residues 60 to 65 (GIVNWQ) (Figure S2).

**Figure 1:**
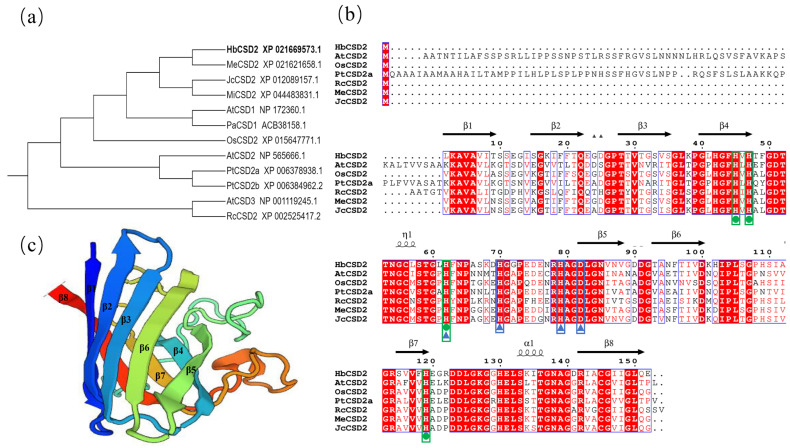
Multiple sequence alignment and phylogenetic tree analysis of Cu/ZnSOD proteins from different species and 3D structure construction of the HbCSD2 protein. (**a**) Phylogenetic tree analysis of CSD proteins from different species; Me, *Manihot esculenta*; Jc, *Jatropha curcas*; Mi, *Mangifera indica*; At, *Arabidopsis thaliana*; Pa, *Potentilla atrosanguinea*; Os, *Oryza sativa*; Pt, *Populus trichocarpa*; Rc, *Ricinus communis*. (**b**) Multiple sequence alignment analysis; green spheres represent Cu^2+^-binding sites; blue triangles represent Zn^2+^-binding sites. (**c**) Predicted 3D structure of the HbCSD2 protein.

Multiple sequence alignments of the HbCSD2 protein with the Cu/ZnSOD proteins from other species which revealed high homology with JcCSD2 (XP_012089157.1) and MeCSD2 (XP_021621658.1), at 80.26% and 79.61%, respectively, as well as with *Potentilla atrosanguinea* PaCSD1 (ACB38158.1), at 78.95%. The homologies of HbCSD2 with the woody model plant poplar PtCSD2a and PtCSD2b were 41.10% and 42.79%, respectively; with *Arabidopsis* AtCSD2 (NP_565666.1), it was 43.06%; and with rice OsCSD2 (LOC4340091), it was 73.68% (Figure 1b). The 3D structure of the HbCSD2 protein was constructed by homology modeling using the SWISS-MODEL online tool and visualized by the ESPript3.0 online tool. The results showed that the HbCSD2 protein consists of eight β-sheets and a small number of α-helices. Furthermore, HbCSD2 contains conserved Cu^2+^- and Zn^2+^-binding sites, where Cu^2+^ coordinates with His residues at positions 45, 47, 62, and 119, while Zn^2+^ binds to His residues at positions 62, 70, and 79, as well as to Asp-82 (Figure 1c). Phylogenetic tree analysis revealed that HbCSD2 was most closely related to the cassava MeCSD2, followed by JcCSD2, which is consistent with the results of multiple sequence alignment (Figure 1a).

### 2.2. Subcellular Localization Analysis

**Figure 2:**
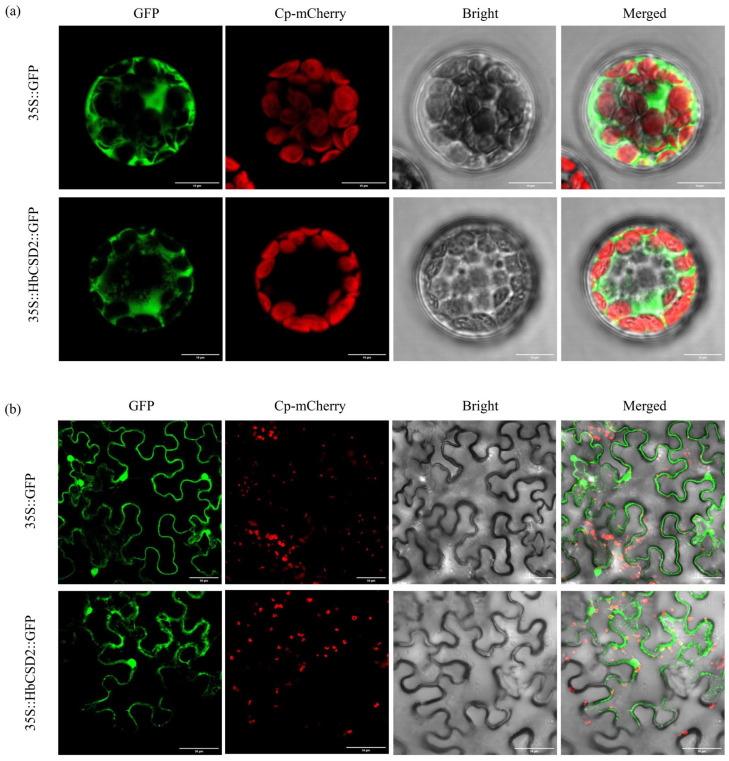
Subcellular localization analysis of the HbCSD2 protein. (**a**) Subcellular localization of the HbCSD2 protein in *Arabidopsis* protoplasts. (**b**) Subcellular localization of the HbCSD2 protein in tobacco leaves. GFP, Green fluorescent protein; Cp-mCherry, mCherry-labeled chloroplast marker. Scale bar, 10 μm.

To determine the subcellular localization of HbCSD2 protein, 35S-HbCSD2-GFP and 35S- GFP were expressed in *Arabidopsis* protoplasts. Images were captured and compared using a confocal microscope. The results are presented in Figure 2. The green fluorescence signal from the *Arabidopsis* protoplasts transiently expressing 35S-GFP was observed throughout the entire cell except for the chloroplasts, whereas the green fluorescence signal from the *Arabidopsis* protoplasts transiently expressing 35S-HbCSD2-GFP was detected exclusively in the cytoplasm and nucleus and did not colocalize with the chloroplast marker (Figure 2a). These results indicated that HbCSD2 protein is localized in the cytoplasm and the nucleus. To further confirm the localization of the HbCSD2 protein, it was transiently expressed in tobacco leaf epidermal cells using the *Agrobacterium*-mediated method [25]. The results indicated that HbCSD2 was localized in the cytoplasm and nucleus (Figure 2b), which is consistent with the subcellular localization results in *Arabidopsis* protoplasts.

### 2.3. Overexpression of HbCSD2 Enhances Cold Tolerance in Transgenic Arabidopsis

**Figure 3:**
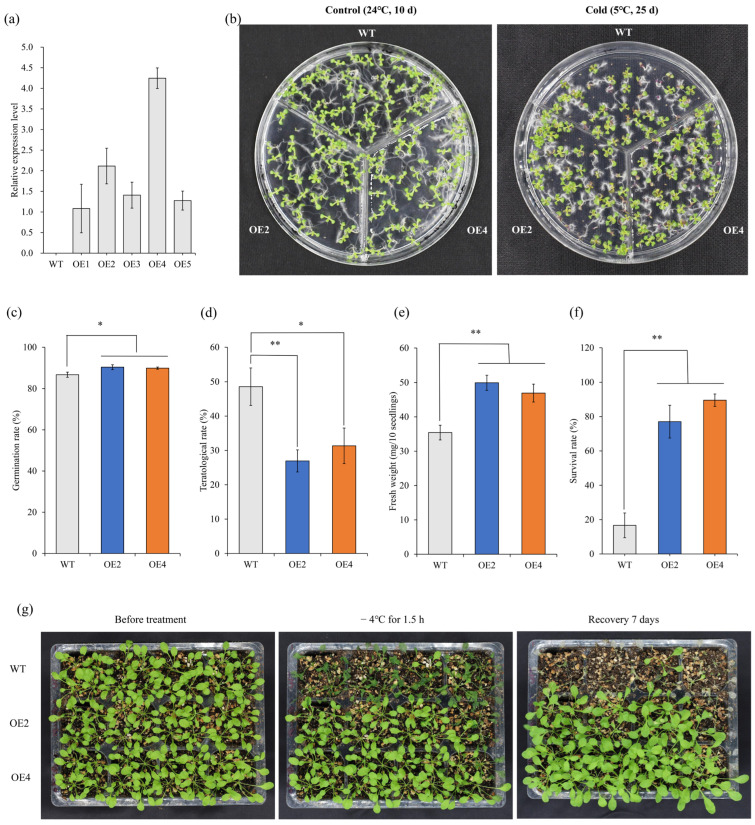
Overexpression of *HbCSD2* enhanced cold tolerance in transgenic *Arabidopsis thaliana.* (**a**) The expression analysis of *HbCSD2* gene in transgenic *Arabidopsis thaliana*. (**b**) Seed germination and phenotypic analysis of transgenic and WT lines under cold (5 °C) stress. (**c**) Germination rate. (**d**) Teratological rate. (**e**) Fresh weight. (**f**) Survival rate of *HbCSD2* transgenic and WT *Arabidopsis thaliana* under low-temperature (−4 °C) stress treatment. (**g**) Phenotypic analysis of transgenic and WT lines under low-temperature (−4 °C) stress treatment. The data are presented as the means ± standard deviations (n = 3). The significance of differences was analyzed using *t*-test, * indicates *p* ≤ 0.05, and ** indicates *p* ≤ 0.01.

In this study, the full-length CDS of *HbCSD2* was cloned and inserted into the pCAMBIA1302 plant expression vector and transformed into the *Arabidopsis* genome using the *Agrobacterium* infection method. Five positive transgenic *Arabidopsis* lines were obtained by hygromycin resistance screening and PCR detection (Figure S3). The transcription levels of *HbCSD2* in T3 generation plants were detected using quantitative real-time PCR (Figure 3a).

To investigate the cold tolerance function of the *HbCSD2* gene, the phenotype, germination rate, malformed seedling rate, and fresh weight were recorded for the T3 transgenic *Arabidopsis* lines (OE2 and OE4) and WT seeds sown in the 1/2 MS medium and cultured at 5 °C for 25 days, 24 °C for 10 d as control. The results revealed that the germination rates of the OE2 and OE4 transgenic lines were 90.42% and 89.94%, respectively, which were significantly greater than that of the WT (86.75%) (Figure 3b,c) under cold condition. The malformed seedling rates of OE2 and OE4 were 26.94% and 31.34%, respectively, which were significantly lower than that of the WT (48.56%) (Figure 3b,d). Additionally, the fresh weights per 10 seedlings of OE2 and OE4 were 49.93 and 46.93 mg, respectively, which were also significantly greater than that of the WT (35.43 mg) (Figure 3e). These findings indicate that the heterologous expression of *HbCSD2* significantly enhances the cold tolerance of transgenic *Arabidopsis*. To further verify the cold tolerance function of the *HbCSD2* gene, 24-day-old transgenic *Arabidopsis* lines (OE2 and OE4) and WT seedlings were subjected to −4 °C treatment for 1.5 h and then allowed to recover at 24 °C for 7 d. The results revealed that the wilting degree of the transgenic lines was lower than that of the WT (Figure 3g), and the survival rates of the transgenic lines OE2 and OE-4 were 77.08% and 89.58%, respectively, which were significantly greater than those of the WT (16.67%) (Figure 3f). These results suggest that overexpression of the *HbCSD2* gene markedly enhances the cold tolerance of transgenic *Arabidopsis*.

### 2.4. Physiological Response of HbCSD2 Transgenic Arabidopsis Plants Under Cold Stress

**Figure 4:**
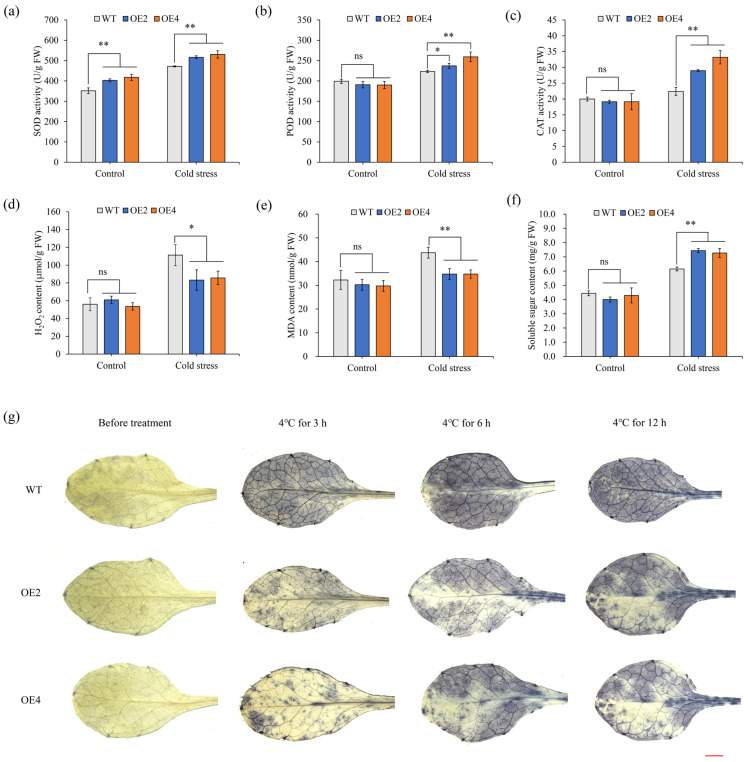
Physiological responses in *HbCSD2* transgenic *Arabidopsis* plants under cold stress. (**a**) SOD activity. (**b**) POD activity. (**c**) CAT activity. (**d**) H_2_O_2_ content. (**e**) MDA content. (**f**) Soluble sugar content. (**g**) NBT staining was performed on Col-0 and transgenic lines subjected to 4 °C cold stress for 3, 6, and 12 h to detect O_2_•^−^ production in leaves under cold stress, 24 °C group as the control. Bars, 2.0 mm. The data are presented as the means ± standard deviations (n = 3). The significance of differences was analyzed by *t*-test, * indicates *p* ≤ 0.05, ** indicates *p* ≤ 0.01, and ns indicates no significance.

To evaluate the ability of the plants to scavenge ROS, we measured the activities of the SOD, POD, and CAT antioxidant enzymes. The results revealed that under normal growth conditions, the SOD activity of the transgenic *Arabidopsis* plants was significantly greater than that of the WT plants, while there was no significant change in the CAT and POD activities (Figure 4a–c). After cold stress, the SOD, CAT, and POD activities of the transgenic lines were significantly greater than those of the WT (Figure 4a–c).

To further investigate the role of *HbCSD2* in low-temperature resistance and ROS scavenging, we measured the H_2_O_2_ content. The results revealed that under normal conditions, the H_2_O_2_ contents in both transgenic *Arabidopsis* and WT plants remained relatively stable and were not significantly different. However, following cold stress treatment, the H_2_O_2_ content in the transgenic *Arabidopsis* seedlings was significantly lower than that in the WT seedlings (Figure 4d). In addition, we assayed O_2_•^−^ accumulation in *HbCSD2* transgenic plants using nitro blue tetrazolium (NBT) histochemical staining after cold (4 °C for 3, 6, and 12 h) treatment. The results (Figure 4g) indicated that under normal growth conditions, the leaves from all the plant lines exhibited light staining, with no notable differences observed between the transgenic *Arabidopsis* and WT plants. In contrast, after cold stress treatment, the staining intensity of all the plants increased with increasing treatment time. Nevertheless, the overall staining intensity in the transgenic lines was still lower than that of the WT plants (Figure 4g). These results suggested that the transgenic *Arabidopsis* lines experienced less ROS accumulation and cell damage under cold stress. We also measured the levels of malondialdehyde (MDA) and soluble sugars. The results revealed that under normal culture conditions, there were no significant differences in the levels of MDA and soluble sugars between the transgenic and WT *Arabidopsis* plants. However, following cold stress treatment, the MDA content in transgenic *Arabidopsis* plants was significantly lower than that of the WT plants (Figure 4e), whereas soluble sugar levels were markedly higher compared to those of the WT plants (Figure 4f).

## 3. Discussion

When plants are exposed to low-temperature stress, the cellular balance of ROS is disrupted, leading to a significant accumulation of radicals. Excessive ROS accumulation can cause membrane lipid peroxidation, structural damage to proteins and nucleic acids, and disturbances in metabolic processes. In severe cases, it may result in cell death, impair plant growth and development, and ultimately reduce yield [6,26,27]. Superoxide dismutase (SOD), as the first line of defense in the enzymatic antioxidant system, catalyzes the dismutation of O_2_•^−^ into H_2_O_2_ and O_2_ [5,9,10].

Cu/ZnSOD is one of the SOD subfamilies, and its members typically exist as homodimers, with each subunit containing a Cu^2+^ and Zn^2+^ ion [28]. In this study, a *Cu/ZnSOD* (*HbCSD2*) gene was successfully cloned from the rubber tree. The open reading frame (ORF) of *HbCSD2* is 459 bp long and encodes a 152-amino-acid-long protein. Compared with the HbCSD2 protein sequence (XP_021669573.1) [29], the deduced amino acid sequence contains a minor deletion spanning residues 60 to 65 (GIVNWQ) (Figure S2). For further verification, we compared the HbCSD2 protein sequence (KAF2287930.1) identified from another rubber tree (clone, GT1) genome [30], and the results showed that the peptide fragment was consistent with the sequence deduced by PCR cloning. These results suggest that the current annotation of HbCSD2 in the rubber tree (7-33-97) genome may be inaccurate, or that the difference in this small peptide may be due to natural variation among different rubber tree varieties. These results will further improve genome annotation.

Protein structure analysis revealed that HbCSD2 contains Cu^2+^- and Zn^2+^-binding sites. Specifically, Cu^2+^ binds to His-45, His- 47, His-62, and His-119 residues, while Zn^2+^ binds to His-62, His-70, and His-79 and Asp-82 residues (Figure 1), which is consistent with previous studies [31,32,33,34]. HbCSD2 protein consists of eight β-sheets and a small number of α-helices (Figure 1), which is consistent with previous studies [33,34,35]. It can be concluded that the Cu^2+^- and Zn^2+^-binding sites of the Cu/ZnSOD protein are highly conserved across various plant species. Previous studies have demonstrated that Cu/ZnSOD proteins are localized in different subcellular compartments, including chloroplasts, the cytoplasm, and mitochondria. For example, the populus PagSOD2a protein is targeted in the chloroplasts [15], whereas the wheat Cu/ZnSOD protein is localized in the cytoplasm, chloroplasts, and mitochondria [36]. To identify the subcellular localization of the HbCSD2 protein, we constructed a fusion expression vector containing HbCSD2 and GFP and transiently expressed in *Arabidopsis* protoplasts and tobacco leaf epidermal cells. These results revealed that the HbCSD2 protein is localized in the cytoplasm and nucleus (Figure 2), consistent with the previous prediction result [24] and previous research results [36].

Numerous studies have demonstrated that Cu/ZnSOD plays a crucial role in plant responses to cold stress. For example, under cold stress (4 °C), the *CsSOD* genes in the tea plants were upregulated, except for *CsFSD2*, whose expression was suppressed [37]. In *Salvia miltiorrhiza*, *SmCSD2* and *SmMSD1* are significantly upregulated under cold stress [38]. Most *CmSOD* genes in melon and *CiSOD* genes were significantly upregulated under cold stress treatment [39]. In *Dendrobium catenatum*, almost all *DcaSOD* genes, with the exception of *DcaFSD2*, are significantly upregulated under cold stress [40]. Additionally, overexpression of the SOD gene improved the cold tolerance of the transgenic plants. For example, under cold stress, the expression of SOD genes, the SOD activity, and the chlorophyll contents in *AtSOD* and *CmSOD* transgenic *Arabidopsis* lines were significantly greater than those in the WT *Arabidopsis* plants. Moreover, the expression of the cold-related gene *AtCBF2* and the drought-related transcription factor *AtRD29A/B* was activated, and the *Arabidopsis* seedlings overexpressing these genes presented increased cold tolerance compared with that of the WT plants [17]. Xu et al. [41] reported that when *MeCu/ZnSOD* and *MeCAT1* were cotransformed into cassava, the SOD and CAT activities of the transgenic lines increased significantly compared with those of the WT lines under cold stress, and the cold tolerance of the transgenic lines was significantly increased. In conclusion, many studies have shown that the overexpression of the SOD gene can significantly enhance the tolerance of crops to cold stress via enhancing ROS scavenging. Therefore, genetic engineering of the SOD functional gene is a feasible molecular breeding strategy for enhancing crop stress resistance.

**Figure 5:**
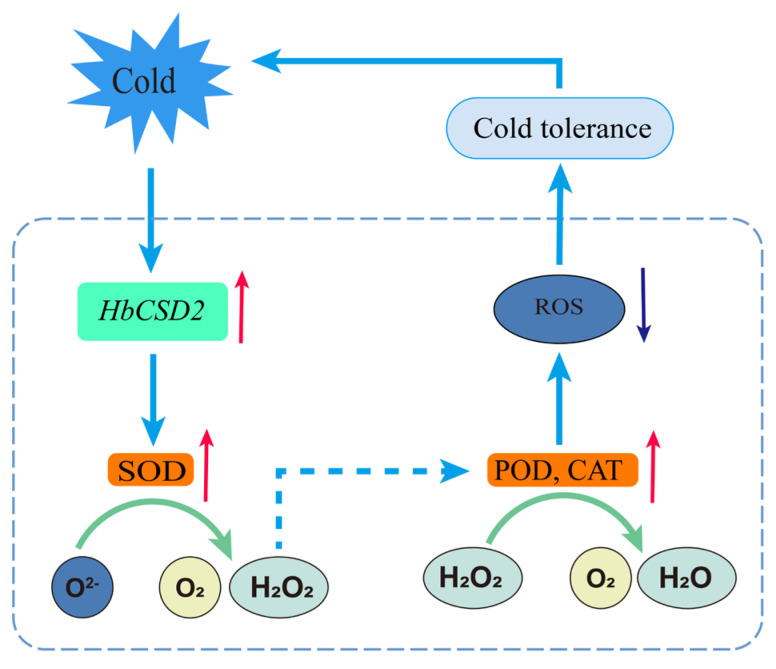
A possible model of *HbCSD2* in response to cold stress. Cold stress induces the expression of the *HbCSD2* gene, which promotes the catalytic dismutation of O_2_•^−^ into H_2_O_2_ and O_2_; the subsequent H_2_O_2_ accumulation further upregulates CAT activity, thereby enhancing the decomposition of H_2_O_2_ into H_2_O and O_2_. These changes effectively reduce ROS levels, thereby contributing to enhanced plant tolerance to cold stress. CSD represents Cu/ZnSOD; red arrows indicate increase; blue arrows indicate decrease; cyan arrows indicate the reaction direction; dash line arrow indicates induction direction; green arrows indicate the catalytic direction.

The rubber trees growing in nontraditional rubber-planting regions at the northern edge of the tropics are frequently exposed to waves of low temperature during winter, resulting in constant low-temperature stress. Low-temperature damage has become a major environmental constraint on the sustainable development of the natural rubber industry. Previous studies demonstrated that the expression of the *HbCSD2* gene was significantly upregulated under low-temperature stress and remained at a high level [24], suggesting that *HbCSD2* may play an important role in the response to low-temperature stress. Therefore, to further explore the biological function of the *HbCSD2* gene under low-temperature stress, we heterologously expressed it in *Arabidopsis* and evaluated the cold resistance of the resulting transgenic lines. The results revealed that the overexpression of the *HbCSD2* gene significantly improved the cold tolerance of the transgenic lines (Figure 3). In addition, under low-temperature stress, the SOD, CAT, and POD activities of the transgenic lines were significantly greater than those of the WT (Figure 4), and the contents of H_2_O_2_ and O_2_•^−^ contents were significantly lower than those of the WT (Figure 4). Based on the above results, we speculated a proposed model for explaining the regulatory mechanism of *HbCSD2*-meidated cold stress response, that is, low-temperature stress may increase SOD activity by inducing the expression of *HbCSD2* gene in the rubber tree, thereby promoting SOD catalyzed dissimilation of O_2_•^−^ into H_2_O_2_ and O_2_. The accumulation of H_2_O_2_ induces an increase in CAT and POD activities, which in turn increases the ability of H_2_O_2_ to decompose into H_2_O and O_2_. Through the synergistic effect of this antioxidant system, the ROS level in the cells decreases, thereby enhancing the cold tolerance of the rubber trees (Figure 5).

## 4. Materials and Methods

### 4.1. Plant Materials and Treatments

The seedlings of cold-resistant rubber tree cultivar GT1 bud-grafting clone were used as experimental materials and cultivated at the experimental base of the Yunnan Tropical Crop Science Research Institute (Jinghong, Yunnan, China). Uniformly growing GT1 bud-grafting clone seedlings with first growth unit leaves were in the stable growth phase were selected and transferred to an artificial climate chamber. These seedlings were acclimated for 48 h under a 16 h light/8 h dark photoperiod, 28 °C temperature, and 80 ± 5% relative humidity. For the low-temperature stress treatment, the temperature was adjusted to 4 °C (the temperature was reduced from 28 °C to 4 °C within 15 min); seedlings without any stress treatment were used as the controls [24,42,43]. Leaves were collected at 0, 1, 3, 6, 12, 24, and 48 h after the low-temperature stress treatment. One leaf was collected from each plant, and the leaves from five plants were mixed into one sample. After this, the samples were quickly placed in liquid nitrogen for freezing and stored at −80 °C.

### 4.2. RNA Extraction and cDNA Synthesis

Total RNA was extracted from the samples using a plant RNA extraction kit (DP441, Tiangen, Beijing, China). To ensure enrichment of the low-temperature-responsive transcripts in the RNA sample, 5 μL of RNA from different time points in the stress treatment was pooled, and 2 μg of RNA was subsequently reverse-transcribed into first-strand cDNA using a reverse transcription kit (K1622, Thermo Fisher Scientific, Waltham, MA, USA).

### 4.3. HbCSD2 Cloning and Sequence Analysis

The specific primers *HbCSD2-F*/*HbCSD2-R* were designed based on the CDS sequence of the *HbCSD2* (NCBI Accession: XM_021813881.1) gene derived from the rubber tree genome database (Table S1). PCR amplification was performed using the rubber tree GT1 leaf cDNA as a template. Then, the target gene was subsequently cloned and inserted into the vector (CT111-01, TRNAS, Beijing, China) using T4-DNA polymerase (FL101-01, TRNAS, Beijing, China). Colony PCR was performed using M13F/M13R primers (Table S1) in a 20 µL reaction mixture containing 2×Taq primer mixture (10 µL), M13F (1.0 µL), M13R (1.0 µL), bacterial solution (1.0 µL), and ddH_2_O (7.0 µL). The PCR reaction conditions were as follows: predenaturation at 95 °C for 5 min, followed by 35 cycles of denaturation at 95 °C for 10 s, annealing at 60 °C for 20 s, and extension at 72 °C for 15 s. Three positive clones were subsequently selected for sequencing (Shengong, Shanghai, China). The sequencing results were aligned with the CDS of the *HbCSD2* gene to confirm the accuracy of the cloned fragment.

The tertiary structure of the HbCSD2 protein was predicted using homology modeling via the SWISS-MODEL online server. Homologous CSD proteins from other species, including those from the model plants *Arabidopsis thaliana* and *Oryza sativa*; rubber tree-related species, such as *Ricinus communis*, *Jatropha curcas*, *Manihot esculenta*, and *Euphorbia peplus*; and the woody plant model *Populus trichocarpa*, were obtained from NCBI. Phylogenetic analysis was conducted using the neighbor-joining (NJ) algorithm in MEGA11.0 software [44], with bootstrap values set to 1000 replicates. The phylogenetic tree was enhanced using the iTOL online website (https://itol.embl.de/, accessed on 6 March 2025) [45]. Additionally, multiple sequence alignments were carried out using DNAMAN 9.0 software.

### 4.4. Subcellular Localization of the HbCSD2 Protein

The CDS of the *HbCSD2* gene without a stop codon was cloned and inserted into the pCAMBIA1302 vector to generate the fusion expression vector 35S-HbCSD2-GFP (Table S1). In accordance with the methods of previous studies [46,47], *HbCSD2* was transiently expressed in *Arabidopsis* protoplasts, with a 35S-GFP empty vector used as the control, and fluorescence was observed and captured via a laser scanning confocal microscope (FV1000, Olympus, Nagano, Japan). To further determine the subcellular localization of the HbCSD2 protein, *Agrobacterium tumefaciens* containing the 35S-HbCSD2-GFP plasmid and the chloroplast localization marker (mCherry) were coinfiltrated into tobacco leaves, and the 35S-GFP as the control [32].

### 4.5. Transformation of HbCSD2 into Arabidopsis thaliana

The full-length CDS sequence of the *HbCSD2* gene was cloned and inserted into the pCAMBIA1302 plant expression vector (Table S1). Then, the 35S-*HbCSD2* plasmid was transformed into *Agrobacterium tumefaciens* GV3101 (Weidi, Changsha, China), which was then transformed *HbCSD2* gene into *Arabidopsis* using the infection method [25]. Transgenic positive plants were screened in 1/2 MS media containing 50 mg/L hygromycin. The genomic DNA of transgenic *Arabidopsis* was extracted using a high-efficiency plant genomic DNA extraction kit (DP350, Tiangen, Beijing, China), and PCR was performed using this DNA as a template and 35S-F/*HbFSD1*-R as primer to ensure that *HbCSD2* was inserted into the *Arabidopsis* genome. The expression level of *HbCSD2* gene in the T3 transgenic *Arabidopsis* plants were detected by RT-qPCR.

### 4.6. HbCSD2 Enhanced the Cold Tolerance of Transgenic Arabidopsis

Based on the RT-qPCR detection results, two transgenic lines with relatively high expression levels, OE-*HbCSD2*-2 (OE2) and OE-*HbCSD2-4* (OE4), were selected for further study. Transgenic (OE2 and OE4) T3-generation homozygous and WT *Arabidopsis* seeds were sown on 1/2 MS media, vernalized at 4 °C for 72 h, transferred to a light incubator, and cultured at 5 °C under 16 h light/8 h dark cycle conditions. Twenty-five days later, the germination rate, deformation rate, and fresh weight were assessed. Malformed seedlings are defined as those exhibiting partial or complete loss of leaf greenness following seed germination and the malformed seedling rate is the percentage of such seedlings among the total population.

To further verify the role of the *HbCSD2* gene in the response to low-temperature stress, 24-day-old transgenic (OE2 and OE4) and WT seedlings were subjected to a low temperature of −4 °C for 1.5 h. Subsequently, the seedlings were transferred to a light incubator at 24 °C. After seven days, phenotypic changes were recorded, and survival rates were calculated. The experiments were carried out with 16 seedlings per replicate and three independent biological replicates, for a total of 48 seedlings.

### 4.7. Physiological Responses of Transgenic Arabidopsis Under Cold Stress

To understand the physiological response of *HbCSD2* transgenic *Arabidopsis* plants under low-temperature stress. 24-day-old seedlings of transgenic (OE2 and OE4) and WT *Arabidopsis* were kept at 4 °C for 12 h. Seedlings grown at 24 °C, 80 ± 5% relative humidity, and a 16 h light/8 h dark cycle with a photon flux of 250 µmol/m/s were used as the control. The activities of the SOD, CAT, and POD, as well as the levels of malondialdehyde (MDA), hydrogen peroxide (H_2_O_2_), and soluble sugars, were measured according to the kits (Nanjing Jiancheng, Nanjing, China). Three independent biological replicates and each replicate included eight plants. Additionally, the nitroblue tetrazolium chloride (NBT) staining kit (PR1100, Solarbio, Beijing, China) was used to detect O_2_•^−^ accumulation in leaves at 0, 3, 6, and 12 h after low-temperature stress treatment.

### 4.8. Statistical Analysis

All the statistical analyses included three independent biological replicates, each biological replicate with three technical replicates. All the data are presented as the means of triplicate trials, and the standard deviation (SD) was calculated. Significant differences were detected by a *t*-test, * *p* ≤ 0.05, ** *p* ≤ 0.01.

## 5. Conclusions

In this study, a *Cu/ZnSOD* gene (*HbCSD2*) was successfully cloned from the rubber tree. The open reading frame sequence of *HbCSD2* is 459 bp long, encoding a protein containing 152 amino acids. The HbCSD2 protein consists of eight β-sheets and a small number of α-helices. In addition, the HbCSD2 protein possesses conserved Cu^2+^- and Zn^2+^-binding sites, where Cu^2+^ binds to His-45, His-47, His-62, and His-119 sites, and Zn^2+^ binds to His-62, His-70, and His-79 and Asp-82 sites. Subcellular localization analysis revealed that the HbCSD2 protein was localized in the cytoplasm and nucleus. Under low-temperature stress, the seed germination rate, fresh weight, and survival rate of HbCSD2 transgenic *Arabidopsis* plants were significantly greater than those of WT plants, and the seedling deformity rate was significantly lower than that of the WT. In addition, the SOD, CAT, and POD activities and soluble sugar content of the HbCSD2 transgenic *Arabidopsis* plants were significantly greater than those of the WT. The H_2_O_2_ and O_2_•^−^ contents, as well as the accumulation of the membrane lipid peroxidation product MDA, were significantly lower in the transgenic plants than that of WT plants. These results suggest that *HbCDS2* confers cold tolerance to transgenic *Arabidopsis*. This study provides candidate genes and a theoretical basis for cold-resistant molecular breeding in rubber trees, and its functional roles can be further validated in rubber trees in the future.

## Data Availability

The data presented in this study are available on request from the corresponding author.

## Supplementary Materials

The following supporting information can be downloaded at https://www.mdpi.com/article/10.3390/plants14233591/s1, Figure S1. Cloning of *HbCSD2* gene. (a) PCR amplification products of *HbCSD2*. (b) PCR detection of positive bacterial liquid; Figure S2. The *HbCSD2* protein sequence derived from PCR amplification aligned with the corresponding genomic sequence; Figure S3. PCR analysis of *HbCSD2* transgenic *A. thaliana*; M, DL2000 Marker; WT, wild type; 1–5, HbCSD2 transgenic *A. thaliana* lines; Table S1. The primers used for this study.
