# Supplementary material for: Overexpression of Hevea brasiliensis HbCDS2 Gene Enhances Cold Tolerance in Transgenic Arabidopsis
Source: Plants (Basel). 2025 Nov 25;14(23):3591. doi: 10.3390/plants14233591 (PMC12694346; doi:10.3390/plants14233591)
Supplement: Supplementary file 1 [file plants-14-03591-s001.zip › supplementary Figure S1.pdf]

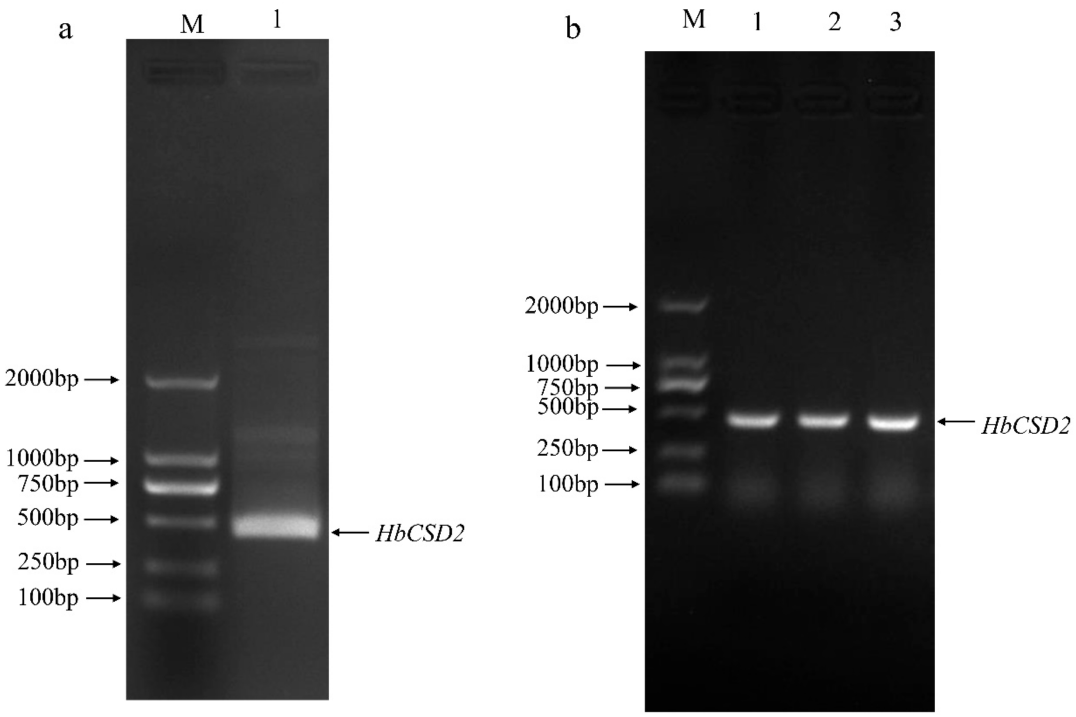

Figure S1 *Cloning of HbCSD2 gene*. (a) PCR amplification products of *HbCSD2*. (b) PCR detection of positive bacterial liquid
