# Supplementary material for: Overexpression of Hevea brasiliensis HbCDS2 Gene Enhances Cold Tolerance in Transgenic Arabidopsis
Source: Plants (Basel). 2025 Nov 25;14(23):3591. doi: 10.3390/plants14233591 (PMC12694346; doi:10.3390/plants14233591)
Supplement: Supplementary file 1 [file plants-14-03591-s001.zip › supplementary Figure S2.pdf]

```
                1         10        20        30        40        50        60
HbCSD2_Genome   MLKAVAVITSSEGISGKIFFTQEGDGPTTVTGSVSGLKPGLHGFHVHTFGDTTNGCLSTG
HbCSD2_PCR      MLKAVAVITSSEGISGKIFFTQEGDGPTTVTGSVSGLKPGLHGFHVHTFGDTTNGCLST.

                         70        80        90       100       110       120
HbCSD2_Genome   IVNWQGLHFNPASKDHGGPEDENRHAGDLGNVNVGDDGTANFTIVDKHIPLSGPHSIAGR
HbCSD2_PCR      .....GLHFNPASKDHGGPEDENRHAGDLGNVNVGDDGTANFTIVDKHIPLSGPHSIAGR

                       130       140       150
HbCSD2_Genome   SVVFHEGRDDLGKGGHELSKITGNAGDRIACGIIGLQE
HbCSD2_PCR      SVVFHEGRDDLGKGGHELSKITGNAGDRIACGIIGLQE
```

Figure S2 The HbCSD2 protein sequence derived from PCR amplification aligned with the corresponding genomic sequence.
