# Supplementary material for: Overexpression of Hevea brasiliensis HbCDS2 Gene Enhances Cold Tolerance in Transgenic Arabidopsis
Source: Plants (Basel). 2025 Nov 25;14(23):3591. doi: 10.3390/plants14233591 (PMC12694346; doi:10.3390/plants14233591)
Supplement: Supplementary file 1 [file plants-14-03591-s001.zip › supplementary Table S1.pdf]

Table S1. The primers used for this study

Primer name	primer sequences (5′-3′)	Usage
HbCSD2-F	ATGTTGAAGGCCGTTGC	Full-length CDS cloning
HbCSD2-R	CTATTCTTGCAAACCAATGATAC	
M13-F	GTAAAACGACGGCCAGT	Positive clone plasmid detection
M13-R	CAGGAAACAGCTATGAC	
35S-F	CACGGGGGACTCTTGCCACC	Positive clone plasmid detection
eGFP-cx	GACACGCTGAACTTGTGG	
1302-HbCSD2-F	CATGCCATGGATGTTGAAGGCCGTTGC	Transgenic Arabidopsis
1302-HbCSD2-R	GGACTAGTCTATTCTTGCAAACCAATGATAC	
HbCSD2-GFP-F	ACTAGGGTCTCGCACCATGTTGAAGGCCGTTGCTGTTATTAC	Subcellular localization analysis
HbCSD2-GFP-R	ACTAGGGTCTCTCGCCTTCTTGCAAACCAATGATACCACATGC	
